# GABA_A_ receptor availability relates to emotion-induced BOLD responses in the medial prefrontal cortex: simultaneous fMRI/PET with [^11^C]flumazenil

**DOI:** 10.3389/fnins.2023.1027697

**Published:** 2023-09-12

**Authors:** Alexander Heinzel, Jörg Mauler, Hans Herzog, Frank Boers, Felix M. Mottaghy, Karl-Josef Langen, Jürgen Scheins, Christoph Lerche, Bernd Neumaier, Georg Northoff, N. Jon Shah

**Affiliations:** ^1^Institute of Neuroscience and Medicine – 4, Forschungszentrum Jülich, Jülich, Germany; ^2^Department of Nuclear Medicine, Medical Faculty RWTH Aachen, Aachen, Germany; ^3^Department of Nuclear medicine, University Hospital Halle, Halle (Saale), Germany; ^4^Department of Radiology and Nuclear Medicine, Maastricht University Medical Centre, Maastricht, Netherlands; ^5^Institute of Neuroscience and Medicine – 5, Forschungszentrum Jülich, Jülich, Germany; ^6^Mind, Brain Imaging and Neuroethics Research Unit, Institute of Mental Health, Royal Ottawa Mental Health Centre and University of Ottawa, Ottawa, ON, Canada; ^7^Institute of Neuroscience and Medicine – 11, Forschungszentrum Jülich, Jülich, Germany; ^8^JARA – BRAIN – Translational Medicine, Aachen, Germany; ^9^Department of Neurology, RWTH Aachen University, Aachen, Germany

**Keywords:** simultaneous fMRI/PET, GABA_A_ receptor, emotion, [^11^C]flumazenil, BOLD signal, binding potential

## Abstract

**Introduction:**

The fMRI BOLD response to emotional stimuli highlighting the role of the medial prefrontal cortex (MPFC) has been thoroughly investigated. Recently, the relationship between emotion processing and GABA levels has been studied using MPFC proton magnetic resonance spectroscopy (1H-MRS). However, the role of GABAA receptors in the MPFC during emotion processing remains unexplored.

**Methods:**

Using [11C]flumazenil PET, we investigated the relationship between the binding potential of GABAA receptors and emotion processing as measured using simultaneous fMRI BOLD. We hypothesized a correlation between the percent signal change in the BOLD signal and the binding potential of GABAA receptors in the MPFC. In a combined simultaneous fMRI and [11C]flumazenil-PET study, we analyzed the data from 15 healthy subjects using visual emotional stimuli. Our task comprised two types of emotional processing: passive viewing and appraisal. Following the administration of a bolus plus infusion protocol, PET and fMRI data were simultaneously acquired in a hybrid 3 T MR-BrainPET.

**Results:**

We found a differential correlation of BOLD percent signal change with [11C]flumazenil binding potential in the MPFC. Specifically, [11C]flumazenil binding potential in the ventromedial prefrontal cortex (vMPFC) correlated with passive viewing of emotionally valenced pictures. In contrast, the [11C]flumazenil binding potential and the BOLD signal induced by picture appraisal did show a correlation in the paracingulate gyrus.

**Conclusion:**

Our data deliver first evidence for a relationship between MPFC GABAA receptors and emotion processing in the same region. Moreover, we observed that GABAA receptors appear to play different roles in emotion processing in the vMPFC (passive viewing) and paracingulate gyrus (appraisal).

## 1. Introduction

The basis of any brain function results from neural signaling and the associated interplay of neurotransmitters that are organized in large-scale networks. These networks cause fluctuations in the cerebral metabolic rate of oxygen consumption, inducing changes in the blood content of deoxyhaemoglobin, as well as changes in blood flow and volume, representing the basis of the fMRI BOLD signal (Lauritzen et al., 2012). One of the biggest challenges in neuroimaging is to understand how these processes (i.e., neurochemistry and neurophysiology) are related to each other on various functional levels.

Gamma-aminobutyric acid (GABA) is considered the principal inhibitory neurotransmitter in the central nervous system (CNS) (de Leon and Tadi, 2021), and many studies have addressed its influence on neural activity and brain hemodynamic oxygen response on a microscopic level (Lauritzen et al., 2012; Kiemes et al., 2021). However, in order to address this relationship at a macroscopic level, different imaging modalities, such as functional magnetic resonance imaging (fMRI), proton magnetic resonance spectroscopy (^1^H-MRS) and positron emission tomography (PET), have been employed in multimodal imaging studies (Duncan et al., 2014a). To date, studies combining ^1^H-MRS and fMRI have delivered evidence of associations between local GABA levels and fMRI activation in visual processing in the occipital lobe (Kiemes et al., 2021). Moreover, they have found a similar relationship between local MRS-based GABA levels and fMRI activation and emotional processing in the MPFC, and, more predominately, in the anterior cingulate (ACC). Many fMRI studies have demonstrated the role of the MPFC in emotional processing, in particular, in the processing of emotional valences (the emotional dimension referring to the extent to which an emotion is positive or negative) (Phan et al., 2002; Heinzel et al., 2005; Grimm et al., 2006; Willinger et al., 2019). Recently, the processing of different emotional valences has been linked to GABA levels in the ACC (Levar et al., 2017). However, it remains unclear how the local GABA levels are related to the anatomical distribution of GABA receptors.

Receptor imaging with [^11^C]flumazenil using positron emission tomography (PET) is a powerful technique used to probe the binding potential of GABA_A_ receptors in humans non-invasively (Murrell et al., 2020). However, the use of combined fMRI and [^11^C]flumazenil PET in human studies remains rather limited. Furthermore, the existing literature is predominantly based on the less sophisticated technique of serial measurements, in contrast to the more advanced approach employed in this study. Applying aversive electrical stimuli, Hayes et al. (2013) showed that individual differences in sensorimotor cortex signal changes could be predicted by GABA_A_ receptors in the MPFC and sensorimotor cortex. Other combined studies have found that GABA_A_ receptors are involved in differential processing of internally and externally guided awareness (Wiebking et al., 2012) as well as in neural variability modulation (Qin et al., 2018).

In this study, we investigated the relationship of the GABA_A_ receptor binding potential with the percent signal change of the fMRI BOLD signal during emotional valence processing using a unique approach to obtain simultaneous fMRI and [^11^C]flumazenil-PET measurements in healthy participants. We hypothesized that changes in the BOLD percent signal would correlate with the binding potential of GABA_A_ receptors in the MPFC. In order to further investigate the specific type of emotional valence processing, we used different tasks requiring either passive viewing or emotional appraisal. In addition to our hypothesis-driven approach regarding the MPFC, we additionally examined other regions in an exploratory whole-brain approach.

## 2. Materials and methods

### 2.1. Participants

Twenty healthy volunteers participated in the study. None of the participants had a history of neurological or psychiatric disease [assessed by the Mini-International Neuropsychiatry Interview (MINI)] (Sheehan et al., n.d.), and all participants completed the German version of the Beck Depression Inventory (BDI) (Kühner et al., 2007), the German version of the Toronto Alexithymia Scale (Bach et al., 1996), and the Positive and Negative Affect Schedule (PANAS) (Watson et al., 1988). None of the participants were using medication at the time of scanning. Standard MRI exclusion criteria were applied.

All measurements were approved by the local ethics committee/federal authorities and were conducted in accordance with the declaration of Helsinki. All participants gave written informed consent prior to the measurement.

### 2.2. MR and PET data acquisition and reconstruction

A detailed description of the synthesis and administration of [^11^C]flumazenil using a bolus plus infusion scheme, along with the details of data acquisition and reconstruction, can be found in (Mauler et al., 2020). Briefly, the measurements were performed using a Siemens (Erlangen, Germany) hybrid 3 T MR-BrainPET scanner at the Forschungszentrum Jülich, Germany (Herzog et al., 2011). Following the administration of the [^11^C]flumazenil bolus and the initiation of the infusion pump, list-mode data were acquired for 100 min. MR data were acquired concurrently with PET data.

Shortly after a bolus injection with a duration of 3 s, structural, T1-weighted MPRAGE (Magnetization Prepared RApid Gradient Echo) MR data were measured (repetition time TR = 2.25 s, echo time TE = 3.03 ms, in-plane field of view FOV = 256 × 192, resolution 1 mm isotropic, 256 sagittal slices acquired with zero gaps). After 60 min, when the beginning of the steady-state interval was reached, functional scans were performed with an echo-planar imaging (EPI) sequence for 50 min (TR = 3 s, TE = 30 ms, matrix size 64 × 64, in-plane resolution 3 mm, 36 slices in ascending order, slice thickness 3 mm, and inter-slice gap 0.75 mm).

The PET images were iteratively reconstructed using PRESTO (120 iterations) (Scheins et al., 2011, 2015) and a framing of 20 × 300 s. Corrections were included to obtain quantitative images, i.e., for normalization, dead-time, attenuation, scattered and random events (Mauler et al., 2020). In addition, motion correction and registration to the T1-weighted MPRAGE data using the Pmod software package (Version 3.5) was also performed.

### 2.3. Paradigm for fMRI

**Figure 1:**
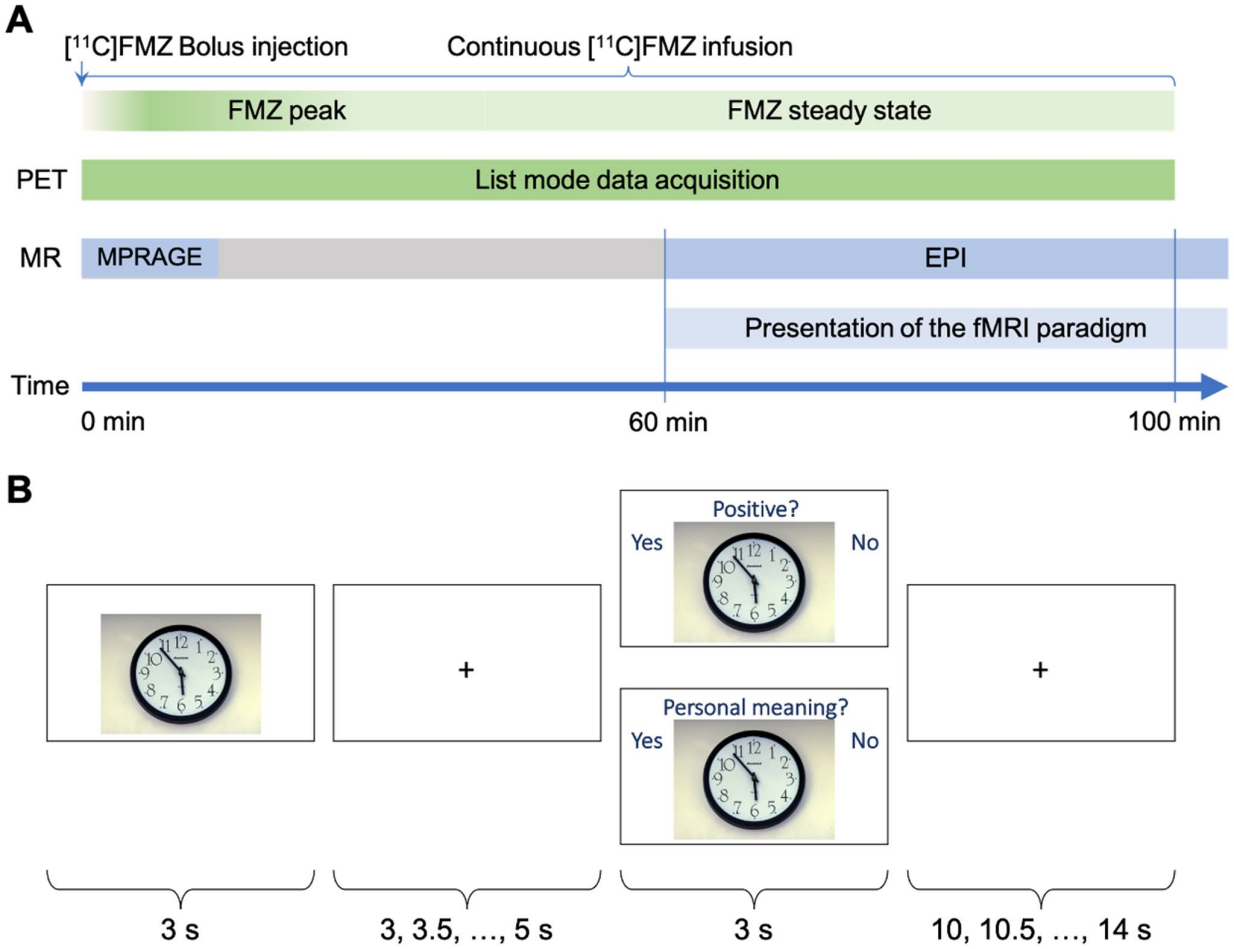
**(A)** Schematic of the experimental design. **(B)** Schematic depiction of a single trial out of the sequence of 114 trials of the fMRI paradigm. The stimulus picture was presented for 3 s, and followed by a fixation cross with variable duration (3–5 s). The stimulus was either shown again with a question referring to the type of judgment and terminated by the fixation cross for 10–14 s or a new picture without question was shown.

Subjects were presented with the fMRI paradigm during the time interval in which the [^11^C]flumazenil steady state was reached (Figure 1A). Since the combined analysis of fMRI and PET data did not focus on binding potential changes at distinct fMRI event times, the fMRI measurement was continued for 10 min after the end of the PET measurement to gain a higher SNR.

The viewing task comprised two types of emotional processing: passive viewing of emotional stimuli and cognitive appraisal of these stimuli with regard to emotional valence (i.e., positive or negative) or self-relevance (i.e., high or low personal relevance of the picture content) (Northoff et al., 2004; Heinzel et al., 2005; Northoff, 2016). Accordingly, participants were asked to first passively view and then to judge visual stimuli from the International Affective Picture System (IAPS) (Lang et al., 2008). Since the focus of this paper is valence processing, the selected IAPS images were matched for arousal levels according to the values stated in Libkuman et al. (2007). Pictures with normative valence ratings ranging between four and six were categorized as neutral, while those with ratings lower than four were considered negative, and those with ratings above six were treated as positive (Grühn and Scheibe, 2008; Gong and Wang, 2016). To this end, 64 emotional (38 negative) stimuli and 50 neutral stimuli were selected for inclusion, with mean arousal values of 3.70 for neutral stimuli and 3.77 for emotional stimuli (negative 3.68; positive 3.90). The mean valence values of the negative stimuli were 2.53, 5.04 for neutral stimuli, and 6.64 for positive stimuli. A schematic representation of the fMRI task is shown in Figure 1B. The IAPS pictures were presented for 3 s (passive viewing), followed by a fixation cross with a variable duration (3.0, 3.5, 4.0, 4.5, and 5.0). Subsequently, the same IAPS picture was presented for a second time, but this time with written instructions indicating the type of judgment (i.e., emotional valence or self-relevance each). The trial was terminated by the presentation of the fixation cross with a variable duration (10.0, 10.5, 11.0, 11.5, 12.0, 12.5, 13.0, 13.5, and 14.0). Altogether, 94 of these trials were presented (47 for each type of judgment) to each participant. In addition, in 20 trials, the passive viewing task was not followed by a judgment task but by another passive viewing task with a different IAPS picture. The judgment was performed by pressing a button on a response device (LUMItouch^™^, Photon Control Inc., BC, Canada) with the index finger of the left or right hand. Reaction times were recorded. The fixation cross served as the baseline condition (Newman et al., 2001; Northoff et al., 2004). The different types of IAPS passive viewing and judgment tasks were presented in a randomized order. Before the experimental session, participants familiarized themselves with the paradigm using a test run with different stimuli.

The pictures were generated using the stimulus presentation and response recording tools from the Presentation Version 14.5 (Neurobehavioral Systems, Inc., www.neurobs.com/) software package. During the fMRI measurements, the pictures were presented on a monitor inside the scanner room, and participants viewed the monitor through a mirror positioned on the head coil in a supine position.

The fMRI task-based functional data were preprocessed and analyzed using statistical parametric mapping 12 (SPM12, Wellcome Department of Imaging Neuroscience, London, https://www.fil.ion.ucl.ac.uk/spm). In order to permit magnetic field saturation, the first three volumes of each condition were discarded. The remaining images were realigned to the mean image, slice-time corrected, and co-registered using the individual 3D-MPRAGE images. All images were normalized to the Montreal Neurological Institute (MNI) space, resampled to the resolution of 2 × 2 × 2 mm, and spatially smoothed using a 5 mm full-width-at-half-maximum Gaussian kernel. In order to obtain the signal changes of the hemodynamic response for the subsequent correlation analysis, intra-individual first-level analyses using the general linear model (Friston et al., 1995) were conducted throughout. The design matrix included regressors encoding neutral, negative, and positive IAPS stimuli, as well as for valence judgment and self-relevance judgment. After defining the first-level models, subject-specific activations were calculated. The contrasts were defined such that they corresponded to percent signal change courses in relation to the following events: passive viewing against baseline for negative, positive, neutral and all pictures; picture appraisal against baseline for negative, positive, neutral and all pictures. Voxels showing possible motor activation upon pressing the buttons on the response devices were excluded from the subsequent combined fMRI and PET data analysis. For this purpose, the two-sample t-tests “picture appraisal minus passive viewing” were conducted for every valence class and converted into masks.

The results were then subjected to the combined fMRI and PET data analysis.

#### 2.3.1. Combined fMRI and PET data analysis

The flumazenil data were registered to the MNI space (iso 2 mm) using SPM12, and the uptake C in every voxel was expressed as the non-displaceable binding potential BP_ND_ = (C-C_ref_)/C_ref_ with the activity C_ref_ from the pure white matter compartment of the pons as the reference region. In order to exclude any (potential) effects from the analysis that were unrelated to the experiment, the binding potential was calculated for exactly those timepoints (i.e., for the corresponding PET frames) at which both conditions were fulfilled: the emotional stimulation paradigm was presented and the steady state was reached (SD from mean calculated over the stimulation interval was less than 10%). In addition, the binding potential was calculated for the pre-stimulation frames – however, it was only calculated for those frames in which the signal was within the same mean ± SD range determined for the stimulation interval. This allowed the comparison of the binding potentials before and during emotional stimulation. The PET signal from the reference region, the pons, was exported and numerically decomposed (Mauler et al., 2020) into the contributions from white and grey matter. The uptake in the pure white matter compartment was used as the reference region uptake, C_ref_. The unwanted contribution from uptake in neuronal nuclei was captured in the value of the pure grey matter compartment and discarded. In order to level out any remaining small signal fluctuations, C_ref_ and C were calculated as the mean values over the included frames. This, and the following calculations, were performed by an in-house developed Matlab program.

**Figure 2:**
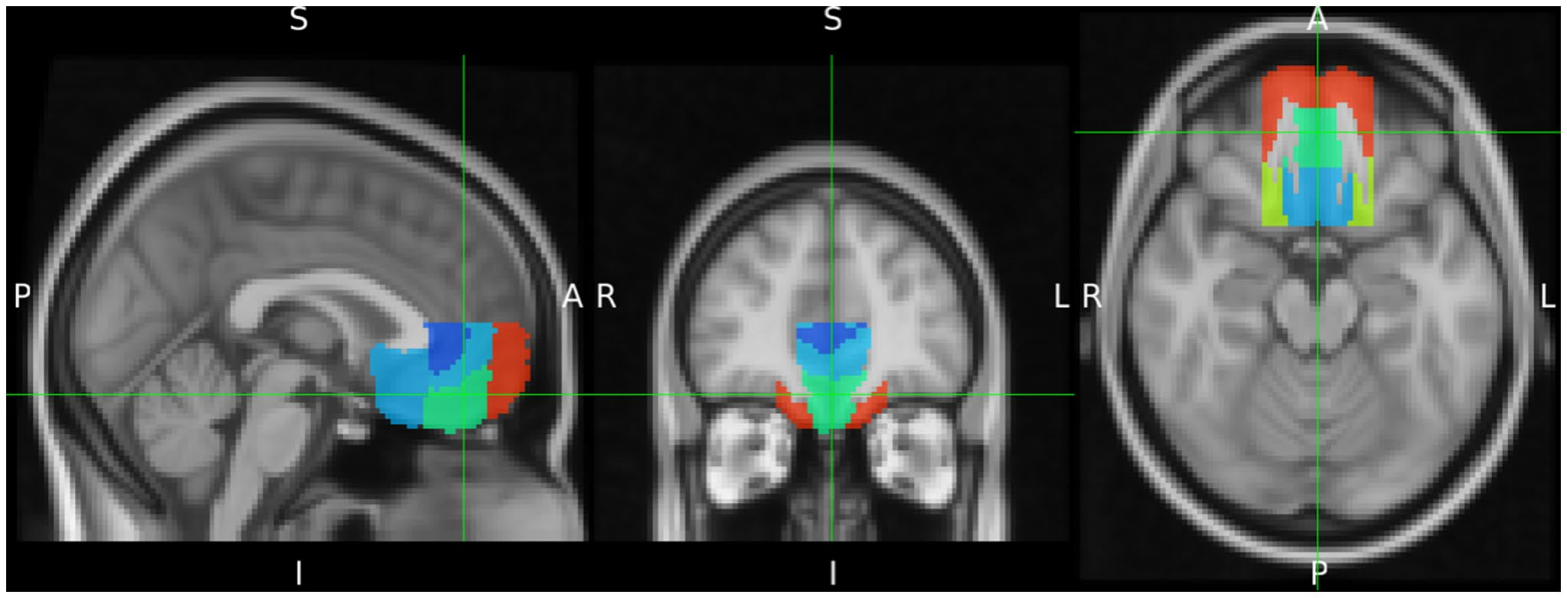
Delineation of the ventromedial prefrontal cortex (vMPFC) composed by regions of the Harvard Oxford Cortical Structures Atlas. It is projected on the MNI iso 2 mm brain. The green cross indicates the Frontal Medial Cortex as part of vMPFC.

Since the definition of the vMPFC varies to some extent between different authors, anatomical VOI masks were defined according to the Harvard-Oxford Cortical Atlas (Desikan et al., 2006). In addition, in order to ensure comparability with other studies, the analysis of the vMPFC was calculated in terms of the regions defined by this atlas (Figure 2). To this end, the vMPFC was defined to include the ventral medial frontal pole, frontal medial cortex (FMC), ventral paracingulate gyrus, ventral anterior cingulate gyrus, and the subcallosal cortex, limited dorsally by the plane z = +10, and laterally by the planes x = ±20 (MNI152 coordinates) (Santos et al., 2011).

To minimize the influence of partial volume effects on the analysis, the partial volume of the grey matter compartment was assessed voxel-wisely by means of the tissue probability maps, which were computed from the MPRAGE data by using SPM12. To match the resolution of the individual tissue probability maps to the effective PET data resolution, the maps were smoothed with an iso 1 mm Gauss kernel following registration to the iso 2 mm MNI space (Mauler et al., 2020). Due to the partial volume effect, voxels with a grey matter content of less than 60% were excluded from further analysis. In addition, voxels indicating potential motor activations were excluded using the masks previously described. The percentage signal change *P* = event signal(s)/mean signal * 100 in the BOLD signal and the flumazenil binding potential values of all remaining voxels from the previously described VOIs were exported. The values from both modalities were subject-individually z-transformed to compensate for the large inter-individual signal differences, VOI-wise averaged and analyzed for the Pearson correlation coefficient between percent signal change and flumazenil binding potential.

## 3. Results

### 3.1. Overview

Twenty participants were measured, of which five subjects were excluded because the acquisition of one modality failed, the measurement was interrupted, or the quality of the steady state was insufficient, leaving data from 15 volunteers (five male) with an average age of 33 ± 13 years [mean ± standard deviation (SD)] for inclusion in the analysis. On average, 383 ± 31 MBq [^11^C]flumazenil was administered per participant using the bolus plus infusion protocol with a mean kbol value of 67 min [53–100 min (minimum – maximum)].

### 3.2. Behavioral results

For the BDI, the mean score was 2.33, with a SD of 2.38. For the TAS-20, the mean score was 48.73, with a SD of 7.01. The mean PANAS score of negative affect was 1.41 (SD 0.4), and the mean score of positive affect was 3.45 (SD 0.51). The reaction times exceeded the maximum time interval of 3 s in 2.9 ± 1.9 out of 94 trials (1–7 trials).

### 3.3. fMRI-PET relationship

The [^11^C]flumazenil binding potential used for the correlation analysis was calculated as the mean of the uptake values over a time interval of 38 ± 8 min (corresponding to 7.6 ± 1.6 frames), beginning at 64 ± 10 min after the bolus injection. The [^11^C]flumazenil binding potential before stimulation was compared to the binding potential upon emotional stimulation and showed no difference. The binding potential before stimulation was calculated from a time interval of 20 ± 12 min (4.1 ± 2.5 frames). The following describes the results of the correlation analysis between the static property of GABA receptor availability (i.e., baseline flumazenil BP_ND_) and the dynamic percent signal change in the BOLD signal as performed on z-transformed data.

#### 3.3.1. Passive viewing

**Figure 3:**
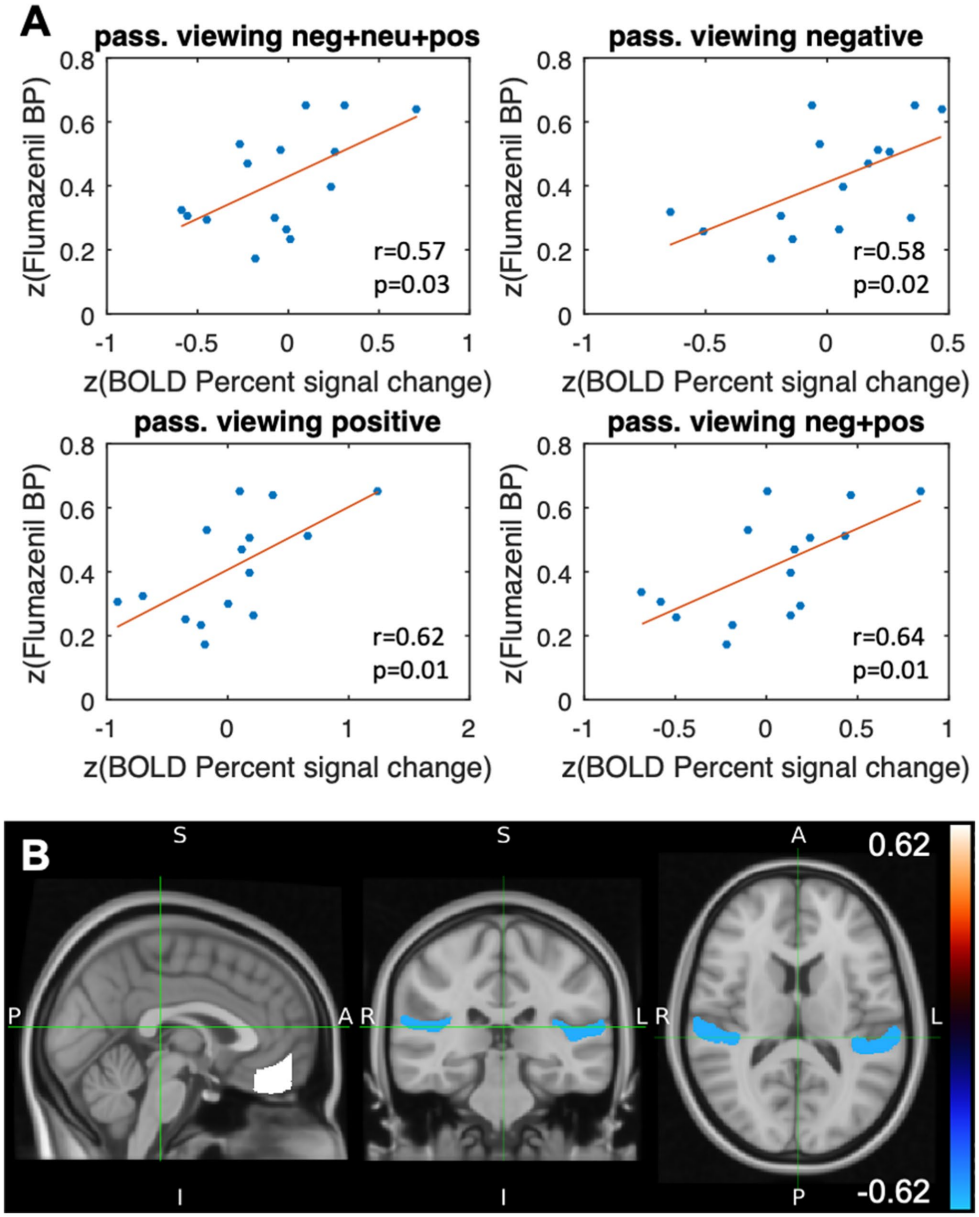
Results after stimulation with the event class “passive viewing.” **(A)** Scatterplots illustrating the VOI-based correlation analyses of the flumazenil binding potential after z-transformation and the z-transformed percent signal change (BOLD) for different types of passive viewing of emotional stimuli (Pearson correlation coefficient and the corresponding *p*-value) in the frontal medial cortex. **(B)** VOI-specific correlation coefficients of the flumazenil binding potential and the percentage signal change in the BOLD signal, both after z-transformation, which were mapped to the planum temporale (*r* = −0.57, *p* = 0.03) and the frontal medial cortex (*r* = 0.62, *p* = 0.01) of the Harvard Oxford Cortical Structures Atlas. Positive stimuli.

The results of the correlation analysis are shown in Table 1A and Figures 3A,B and, in line with our *a priori* hypothesis, indicate that percent signal changes in the BOLD signal in the FMC correlate moderately positively with [^11^C]flumazenil binding potential. The underlying absolute BP_ND_ values (i.e., without z-transformation) in the FMC were 4.34 ± 1.8 (1.51–6.32) (the individual absolute BP_ND_ and BOLD percent signal change values for all subjects are listed in Supplementary Table S1). However, while this was found to be true for passive viewing of emotionally valenced pictures (negative and/or positive), it was not the case for neutral pictures. Considering the vMPFC as a whole, similar results were found for passive viewing of emotionally valenced pictures, and significant correlations (*p* < 0.05) were noted for positive pictures. Thus, the vMPFC, and the FMC in particular, showed a specific moderate correlation between the percent signal change and the [^11^C]flumazenil binding potential while passively viewing emotional stimuli.

**Table 1:** Results from the correlation analyses for the contrasts related to passive viewing of visual stimuli in part A and picture appraisal (i.e., emotional judgment) in part B.

Part A					
	Passive viewing [Pearson correlation coefficient *r* (*p*-value)]				
Regions of medial prefrontal cortex	Negative + Neutral + Positive	Negative	Neutral	Positive	Negative + Positive
Frontal medial cortex	0.57 (0.03)	0.58 (0.02)	–	0.62 (0.01)	0.64 (0.01)
Paracingulate gyrus	–	–	–	–	–
Ventromedial prefrontal cortex	–	–	–	0.55 (0.04)	–
**Other regions**					
Insular Cortex	–	–	–	−0.69 (0.004)	–
Planum Temporale	−0.64 (0.01)	–	−0.55 (0.04)	−0.57 (0.03)	–
Heschl’s gyrus, includes H1 and H2	–	–	–	−0.57 (0.03)	–
Planum Polare	–	–	–	−0.60 (0.02)	–

Part B					
	Picture appraisal [Pearson correlation coefficient *r* (p-value)]				
Regions of medial prefrontal cortex	Negative + Neutral + Positive	Negative	Neutral	Positive	Negative + Positive
Frontal_medial_cortex	–	–	–	–	–
Paracingulate gyrus	0.52 (0.048)	–	0.60 (0.02)	–	–
Ventromedial prefrontal cortex	–	–	–	–	–
**Other regions**					
Heschl’s gyrus, includes H1 and H2	–	0.52 (0.046)	–	–	–
Parietal operculum cortex	–	−0.69 (0.004)	–	–	−0.56 (0.03)
Planum temporale	0.59 (0.02)	0.71 (0.003)	–	–	0.63 (0.01)

The table illustrates the Pearson correlation coefficients r (with *p* < 0.05) of percent signal changes in the BOLD signal with [^11^C]flumazenil binding potential, both after z-transformation. All regions, except the ventromedial prefrontal cortex, refer to the Harvard-Oxford cortical atlas.

The whole-brain analysis revealed other correlations that were not included in our *a priori* hypothesis. These regions comprise the insular cortex, planum temporale, Heschl’s gyrus, and the planum polare. In contrast to the MPFC, these regions all showed negative (rather than positive) correlations in the BOLD signal changes and the [^11^C]flumazenil binding potential (Table 1). The highest absolute correlation coefficients were found for the insular cortex (passive viewing of positive stimuli) and the planum temporale (combined analysis of passive viewing of negative, positive, and neutral stimuli) (Table 1A). The most correlations were found for the passive viewing of positive stimuli.

#### 3.3.2. Picture appraisal

**Figure 4:**
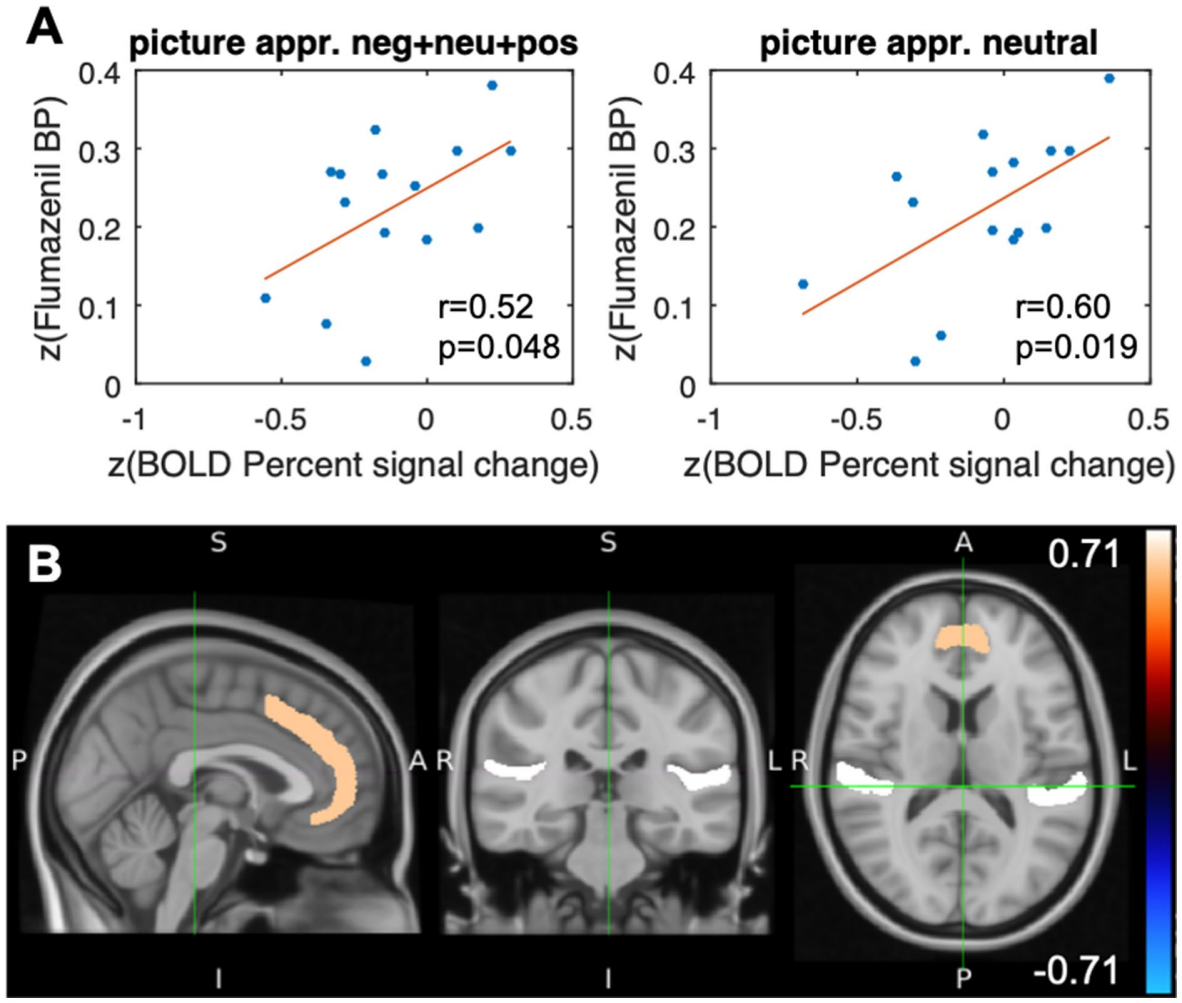
Results after stimulation with the event class “picture appraisal.” **(A)** Scatterplots of the paracingulate gyrus illustrating the Pearson correlation coefficients and the corresponding value of ps of the flumazenil binding potential and the percent signal change (BOLD), both after z-transform, for different appraisals of the stimuli. **(B)** Representation of the VOIs of the Harvard Oxford Cortical Structures Atlas. The color code represents the correlation between the z-transformed flumazenil binding potential and the z-transformed percentage signal change in the BOLD signal in the paracingulate gyrus (*r* = 0.60, *p* = 0.019) and the planum temporale (*r* = 0.71, *p* = 0.003). The subjects assessed neutral and negative visual stimuli in terms of self-relatedness and emotional valence.

The results from the correlation analysis shown in Table 1B and Figures 4A,B demonstrate that changes in the BOLD signal correlate significantly (*p* < 0.05) with [^11^C]flumazenil binding potential in the paracingulate gyrus. The correlations were found to be significant for the combined analysis of picture appraisal of negative, positive, and neutral stimuli, as well as separate analyses of neutral stimuli. The largest moderately sized correlation was found for neutral stimuli. No correlation was found in the vMPFC. The underlying absolute BP_ND_ values (i.e., without z-transformation) in the paracingulate gyrus were 3.99 ± 1.58 (1.4–5.68) (the individual absolute BP_ND_ and BOLD percent signal change values for all subjects are listed in Supplementary Table S2).

The whole-brain analysis revealed additional significant correlations (*p* < 0.05) in the parietal operculum cortex, Heschl’s gyrus, and the planum temporale. In contrast to the results from passive viewing, all regions except the parietal operculum showed positive correlations between the percent change in the BOLD signal and the [^11^C]flumazenil binding potential (Table 1B).

In order to further characterize the type of judgments, correlations of valence-related and self-related judgment were also investigated. A significant positive correlation with the paracingulate gyrus (*r* = 0.52, *p* < 0.05) was found for self-related judgments. The planum temporale showed a positive correlation (*r* = 0.61, *p* < 0.05) for the valence-related judgment task. This suggests, albeit tentatively, that the above-mentioned correlations were driven by emotional valence and the self-specificity of our emotional stimuli.

## 4. Discussion

A differential correlation of the BOLD signal with [^11^C]flumazenil binding potential was found within the MPFC. Furthermore, the binding potential of [^11^C]flumazenil in the vMPFC, and particularly in the FMC, correlated moderately with the passive viewing of negatively and positively valenced pictures. No significant correlation was found with passive viewing of neutrally valenced pictures.

In contrast, although the binding potential of [^11^C]flumazenil in the paracingulate gyrus correlated with the BOLD signal induced by picture appraisal, no such correlation was found in the vMPFC. Moreover, the results indicate that changes in BOLD signal induced by picture appraisal are related to both emotional valence judgment and to self-related judgment.

Various functional imaging studies have demonstrated that the MPFC is commonly activated in emotional processing, including general processes, emotion evaluation, experience, and regulation (Phan et al., 2002; Northoff et al., 2004; Phan et al., 2004; Heinzel et al., 2005; Grimm et al., 2006; Willinger et al., 2019). In addition, studies have shown that the MPFC is specifically involved in the processing of emotional valence (Heinzel et al., 2005; Grimm et al., 2006; Hayes et al., 2014; Yang et al., 2020). The fMRI-PET correlation analysis conducted in this study extends these findings by indicating a specific correlation between visual emotional valence processing and [^11^C]flumazenil binding potential. To date, only ^1^H-MRS has been used to investigate the relationship between GABA levels and emotional processing in the MPFC, and meta-analyses investigating GABA levels in the MPFC have focused predominately on the ACC. The results of these studies have been mixed, with some suggesting a negative relationship with neural activity (Duncan et al., 2014b; Kiemes et al., 2021) and others contradicting this view (Kiemes et al., 2021). Although we did not find a negative correlation between the [^11^C]flumazenil binding potential and the ACC, a moderate positive correlation with the paracingulate gyrus and the FMC was established. However, the role of the experimental setup must be considered when comparing GABA levels resulting from MRS with [^11^C]flumazenil binding potential, as MRS measures global GABA levels (intra- and extra-cellular) generated from the average tissue concentration of GABA, whereas PET permits the detection of alterations in GABA levels in the extracellular space (Frankle et al., 2015). The experimental design used did not measure rapid changes in BP_ND_ under emotional stimulation. Instead, it measured the availability of the GABA receptor and the associated percent signal change in the BOLD signal induced by different types of emotional processing.

In addition, the results from the literature show initial evidence for a negative relationship between GABA levels (measured with ^1^H-MRS) and [^11^C]flumazenil binding potential (Persson et al., 2021) as well as with plasma GABA and [^11^C]flumazenil binding potential (Klumpers et al., 2010). This was interpreted as a form of homeo-static regulation reflected by a regional balance between the transmitter and receptor of the GABA system. Thus, more availability of GABA might be associated with less GABA_A_ receptor availability (i.e., due to downregulation) in the brain (Persson et al., 2021). Therefore, it can be speculated that the positive correlation of GABA_A_ receptor availability and BOLD signal in the vMPFC is associated with less available GABA, as measured with 1H-MRS.

Animal experiment data have shown a positive BOLD response in excitatory neurons, whereas the stimulation of inhibitory neurons resulted in a biphasic response at the stimulation site and a negative BOLD response at downstream sites (Moon et al., 2021). The presented VOI-based analysis captures the dominating correlation but does not differentiate between the spatially intermingled excitatory, inhibitory and downstream neurons in the VOI. This effect may have contributed to the finding of negative correlation coefficients in the planum temporale, Heschl’s gyrus, planum polare and parietal operculum cortex, and the positive correlation coefficients in the regions of the MPFC, which were partially in contrast with literature findings.

Moreover, we identified correlations in two different regions within the MPFC. In contrast to emotional appraisal, which was found to be related to the paracingulate gyrus, significant correlations for passive viewing were located in the vMPFC and, in particular, proved to be moderately large in the FMC. Based on functional imaging studies mainly using fMRI, the vMPFC has been specifically related to emotional experience, which fits in with our results for the task involving passive viewing of emotional stimuli (Heinzel and Northoff, 2009, 2014). As noted above, no correlation was found with regard to the passive viewing of neutral stimuli, indicating that the correlation with [^11^C]flumazenil binding potential in the vMPFC might be specific to the emotional component of picture viewing. Our findings are further supported by previous studies indicating an association between the paracingulate gyrus and reality monitoring, cognitive functions, and the salience network, which is responsible for determining and assigning saliency to events and stimuli (Fornito et al., 2004; Buda et al., 2011; Garrison et al., 2015; Levar et al., 2019). Picture appraisal, as applied in our experimental paradigm, contains cognitive elements in the context of the evaluation of emotional stimuli with regard to valence or self-relatedness.

In addition to our *a priori* hypotheses, the whole-brain analysis revealed that the planum temporale and the Heschl’s gyrus also showed correlations with passive viewing and judgment of the stimuli. More specifically, a negative correlation was found for passive viewing, and a positive correlation was found for emotional appraisal in both regions. The planum temporale has been related to various functions, including visual motion perception and visual memory (Antal et al., 2008; Isato et al., 2021), and, based on our results, it is possible to speculate that it has an even more general role in visual processing, including the visual processing of emotional and neutral stimuli. The association of Heschl’s gyrus with inner speech (Hurlburt et al., 2016) may also indicate that it plays a role in decision-making during emotional judgment. However, since these results are not based on our *a priori* hypotheses, they should be interpreted with caution.

The analysis used the calculation of a modified distribution volume that is determined at tracer equilibrium in order to calculate the [^11^C]flumazenil binding potential (Lassen, 1992). This method was preferred over modeling with a reference tissue model because it was easy to apply – especially with relatively low SNR and when possible changes in the binding potential upon emotional stimulation could not be completely ruled out. However, for comparison with kinetic modeling, data were fitted with the simplified reference tissue model (Lammertsma and Hume, 1996); based on the assumption that modeling the changes in the BP during stimulation is unnecessary) and the linear simplified reference tissue model (Alpert et al., 2003) (results not shown), both of which were published based on a bolus without infusion experiment. The test of the first model revealed that the underlying system of equations was rank deficient and ill-conditioned. The test of the second model showed that it was not able to fit the steady state. Therefore, using these reference tissue models did not result in more robust outcomes than the steady state distribution volume method.

When evaluating the results of this study, some further limitations should also be considered. Perhaps most notably, GABA levels were not assessed using ^1^H-MRS. In order to further investigate the relationship between GABA levels, BOLD response, and [^11^C]flumazenil binding potential in the MPFC, studies should be performed that measure all three parameters. The range of raw BP_ND_ values includes the range of mean values found for [^11^C]flumazenil by other groups (Koeppe et al., 1991; Abadie et al., 1992; Klumpers et al., 2008; Miederer et al., 2009). In contrast to the results from the literature, the binding potential values show a larger standard deviation. These comparisons are only for orientation since the VOIs differ to some extent in position and size. Moreover, other groups frequently carried through kinetic modeling on data acquired immediately after the bolus injection when the SNR is high. Our analysis is based on data that were acquired when the steady state was reached after three half-lives of ^11^C. This may have contributed to the expanded BP range. The literature (Feng et al., 2016) shows elevated standard deviations of the steady state distribution volumes, which results in an increased propagated scatter of BP values. Relatively small deviations in the denominator of the distribution volume ratio cause a large deviation in the result. A possible approach to estimate BP_ND_ with higher accuracy in future studies could be to perform arterial blood sampling so that kinetic modeling with an arterial input function can be applied.

The applied event-related design contained a presentation of the stimuli for 3 s, followed by a relatively long period devoid of stimulus presentation, which – if at all – leads to relatively rapid fluctuations in GABA binding. Given the sensitivity and spatial resolution of the PET scanner used (Herzog et al., 2011), rapid changes in the binding potential after single and short stimuli are not measurable. The analysis focuses on the correlation between the static [^11^C]flumazenil binding potential and the percent signal change in the BOLD signal.

An inherent problem of correlating variables derived from methods with low signal strength, such as fMRI, is that larger sample sizes may be required in order to ensure robust contrast to noise ratio. Large groups of >100 participants have been suggested (Turner et al., 2018). A possible solution to overcome the enormous financial and logistical barriers might be to set up multi-center studies (Duncan et al., 2019).

Finally, our results are based on predefined regions from the Harvard-Oxford atlas. Since this method considers each region as a whole, the exact sizes of the significant clusters inside the predefined regions remain unclear.

## 5. Conclusion

These results provide initial evidence for a direct relationship between MPFC GABA_A_ receptor availability and emotional processing in the same region. Moreover, differential correlations of the BOLD signal and [^11^C]flumazenil binding potential were found for active appraisal and passive viewing of emotional stimuli, indicating the role of the vMPFC in passive viewing and the paracingulate gyrus in appraisal.

## Data availability statement

The datasets presented in this article are not readily available because the ethical approval of the study does not allow the data to be made available to outsiders. Requests to access the datasets should be directed to j.mauler@fz-juelich.de.

## Ethics statement

The studies involving humans were approved by Ethikkommission an der Med. Fakultät der HHU Düsseldorf Gebäude 14.82.01 Moorenstr. 5 D-40225 Düsseldorf Germany. The studies were conducted in accordance with the local legislation and institutional requirements. The participants provided their written informed consent to participate in this study.

## Author contributions

AH, GN, HH, and NS designed the study. AH, JM, HH, JS, FB, CL, FM, BN, and K-JL contributed to data collection and data analysis. AH, JM, NS, and GN drafted the manuscript. All authors contributed to the article and approved the submitted version.

## Funding

The study was funded from the institute’s budget (Institute of Neuroscience and Medicine - 4, Forschungszentrum Jülich (member of the Helmholtz Association), Jülich, Germany), which is funded by federal and state funds. Open access publication funded by the Deutsche Forschungsgemeinschaft (DFG, German Research Foundation) – 491111487.

## Conflict of interest

The authors declare that the research was conducted in the absence of any commercial or financial relationships that could be construed as a potential conflict of interest.

## Publisher’s note

All claims expressed in this article are solely those of the authors and do not necessarily represent those of their affiliated organizations, or those of the publisher, the editors and the reviewers. Any product that may be evaluated in this article, or claim that may be made by its manufacturer, is not guaranteed or endorsed by the publisher.
