# Supplementary material for: GABAA receptor availability relates to emotion-induced BOLD responses in the medial prefrontal cortex: simultaneous fMRI/PET with [11C]flumazenil
Source: Front Neurosci. 2023 Sep 12;17:1027697. doi: 10.3389/fnins.2023.1027697 (PMC10520870; doi:10.3389/fnins.2023.1027697)
Supplement: Supplementary file 1 [file Data_Sheet_1.PDF]

Supplementary Material

1 Supplementary Tables

Tables S1 and S2 show the data underlying Figures 3A and 4A without z-transformation.

Supplementary Table S1: The BOLD percent signal change (PSC) and [^{11}C] flumazenil binding potential (FMZ BP) underlying Figure 3A are presented for all subjects. Four emotional stimuli types (involving basic stimuli with negative (neg), neutral (neu), and positive (pos) valence) led to different percent signal changes in the BOLD signal.

BOLD PSC				FMZ BP
passive viewing neg+neu+pos	passive viewing negative	passive viewing positive	passive viewing neg+pos	
2.88	0.64	0.79	1.43	5.27
0.83	-0.06	0.36	0.30	5.08
-0.36	-0.17	-0.15	-0.33	1.51
-1.74	-0.58	-0.37	-0.94	1.73
-0.62	0.01	-0.04	-0.03	1.63
0.65	0.14	0.15	0.29	5.95
-0.03	0.31	0.15	0.46	4.96
2.50	0.80	0.83	1.63	1.52
0.64	0.28	0.76	1.04	6.32
0.37	0.41	0.13	0.53	4.16
0.64	0.22	0.40	0.62	6.26
-1.00	-0.25	-0.36	-0.62	5.02
-0.10	-0.12	0.34	0.23	5.57
-1.08	-0.48	-0.48	-0.94	4.96
-1.31	-0.24	-0.41	-0.64	5.09

Supplementary Table S2: The BOLD percent signal change (PSC) and [^{11}C] flumazenil binding potential (FMZ BP) for all subjects. These data provide the basis for Figure 4A. The two different emotional stimuli types (involving basic stimuli with negative (neg), neutral (neu), and positive (pos) valence) result in different BOLD percent signal changes.

BOLD PSC		FMZ BP
picture appraisal neg+neu+pos	picture appraisal neutral	
5.85	1.75	4.88
1.34	0.54	4.72
0.51	0.60	1.40
1.30	0.90	1.68
0.76	0.14	1.53
0.81	0.54	5.20
0.66	0.14	4.29
5.75	2.09	1.54
-0.29	0.65	5.55
3.61	1.45	4.31
1.82	0.48	5.68
-1.11	-0.60	4.66
-0.07	0.29	4.99
0.32	0.33	4.93
1.29	0.47	4.57

2 Supplementary Figures

Figures S1 and S2 illustrate the BOLD response to emotional stimulation for a representative case.

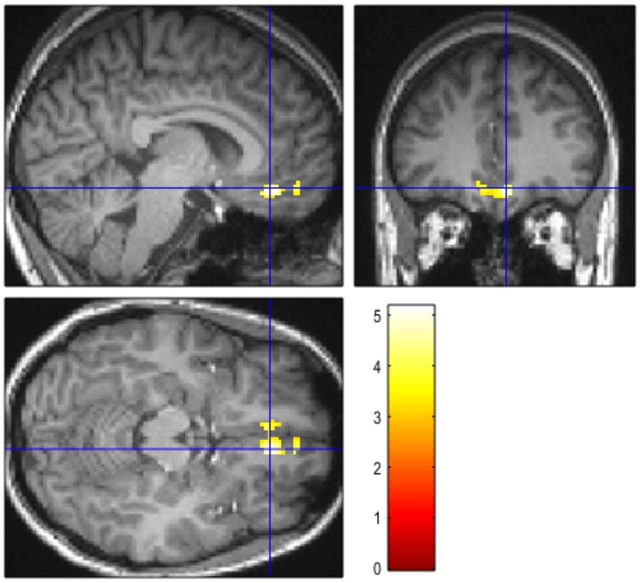

Supplementary Figure S1. Example of an fMRI-result in the frontal medial cortex. When stimulated with the event class "passive viewing of negative and positive stimuli", volunteer 1 showed a clear signal in the frontal medial cortex. Visualisation: using SPM12, the hemodynamic response function was fitted to the BOLD signal changes, and T-statistics were applied ($p < 0.05$). The results were masked with the VOI "frontal medial cortex" of the Harvard Oxford Cortical Structures Atlas and mapped onto the T1-weighted image.

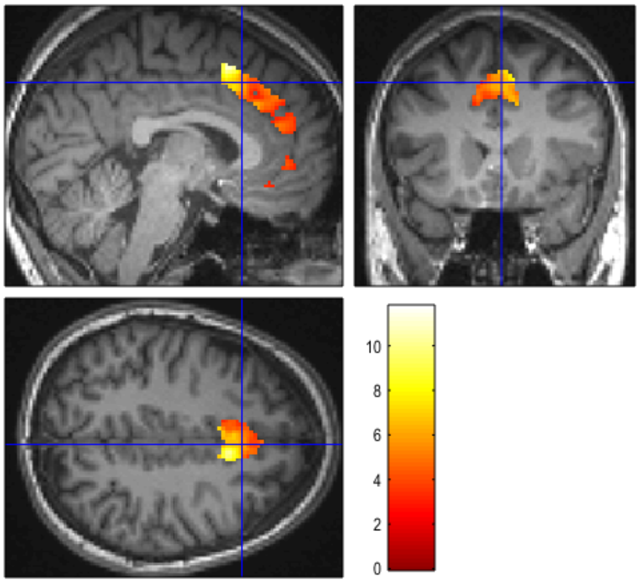

Supplementary Figure S2. Example of an fMRI-result in the paracingulate cortex. The stimulation with the events of the class “picture appraisal of neutral stimuli” led to a strong signal in the paracingulate cortex of volunteer 1. Visualisation: after fitting the hemodynamic response function to the BOLD signal, the T-statistics maps ($p < 0.05$) were masked with the VOI “paracingulate cortex” of the Harvard Oxford Cortical Structures Atlas and mapped onto the T1-weighted image.
